# Expression profile analysis of mycotoxin-related genes in cartilage with endemic osteochondropathy kashin-beck disease

**DOI:** 10.1186/1471-2474-13-130

**Published:** 2012-07-24

**Authors:** Feng Zhang, Xiong Guo, Weizhuo Wang, Shixun Wu, Weijuan Ma, Hua Yan

**Affiliations:** 1 Key Laboratory of Environment and Gene Related Diseases of Ministry Education, Faculty of Public Health, College of Medicine, Xi’an Jiaotong University, Xi’an, Shaanxi, People’s Republic of China, 710061; 2 Department of Orthopedics Surgery, The Second Affiliated Hospital, College of Medicine, Xi’an Jiaotong University, Xi’an, Shaanxi, 710004, People’s Republic of China; 3 National Engineering Research Center for Miniaturized Detection Systems, Northwest University, Xi’an, Shaanxi, 710069, People’s Republic of China

**Keywords:** Kashin-beck disease, Mycotoxins, Microarray

## Abstract

**Background:**

Kashin-Beck Disease (KBD) is an endemic osteochondropathy. Mycotoxins are believed to play an important role in the pathogenesis of KBD. Because the molecular mechanism of mycotoxin-induced cartilage lesions remains unclear, there is not effective treatment for KBD now. To identify key genes involved in the mycotoxin-induced cartilage lesions, we compared the expression profiles of mycotoxin-related genes (MRG) between KBD cartilage and healthy cartilage.

**Methods:**

Total RNA was isolated from cartilage samples, following by being amplified, labeled and hybridized to Agilent human whole genome microarray chip. qRT-PCR was conducted to validate the microarray data. 1,167 MRG were derived from the environmentally related genomic database Toxicogenomics. The microarray data of MRG was subjected to single gene and gene ontology (GO) expression analysis for identifying differently expressed genes and GO.

**Results:**

We identified 7 up-regulated MRG and 2 down-regulated MRG in KBD cartilage, involved in collagen, apoptosis, metabolism and growth & development. GO expression analysis found that 4 apoptosis-related GO and 5 growth & development-related GO were significantly up-regulated in KBD cartilage.

**Conclusions:**

Based on the results of previous and our studies, we suggest that mycotoxins might contribute to the development of KBD through dysfunction of MRG involved in collagen, apoptosis and growth & development in cartilage.

## Background

Kashin-Beck Disease (KBD) is an endemic osteochondropathy characterized by serious articular cartilage necrosis [1]. More than 2.5 million people suffer from KBD and about 30 million people are at the risk of KBD in China [2]. KBD usually occurs in the children aged 3–15 years with patients exhibiting short stature and joint deformities [1,3]. With age, secondary osteoarthritis and deformities of multiple joints will become evident in KBD patients [1,4]. KBD results in significant reduction of patients’ quality of life as well as heavy medical and financial burdens to local governments in China.

Various environmental etiologic hypotheses were proposed for KBD, such as selenium deficiency and cereal contamination by mycotoxins [4-7]. Mycotoxins are believed to play an important role in the pathogenesis of KBD [5]. Previous studies observed significant cytotoxicity of mycotoxins in cartilage [8-11]. However, the molecular mechanism of mycotoxin-induced cartilage lesions in KBD remains unclear, which make it difficult to develop efficient treatments for KBD. Most of current treatments of KBD focus on releasing the pains from secondary osteoarthritis and correcting joint deformities through surgery [12]. Understanding the molecular mechanism of mycotoxin-induced cartilage lesions is the key to develop effective treatments for KBD.

With the rapid development of high-throughput technologies, comparing genome-wide gene expression profiles between case and control groups become possible now. Significantly differentially expressed genes are likely to involve in the development of target diseases, and provide insight for revealing potential pathogenesis. To identify the key genes contributing to mycotoxin-induced cartilage lesions in KBD, we compared the expression profiles of 1,167 mycotoxin-related genes (MRG) between KBD cartilage and healthy cartilage. Both single gene and gene set expression analysis [13,14] were conducted to identify differently expressed MRG and gene ontology(GO). To the best of our knowledge, this study is the first MGR expression analysis of KBD. Our results may help to unravel the molecular mechanism of mycotoxin-induced cartilage lesions in KBD.

## Methods

All studies were approved by the Institutional Review Boards of Xi’an Jiaotong University. Informed-consent documents were written by all KBD patients and the relatives of donors.

### Cartilage sample collection

**Figure 1:**
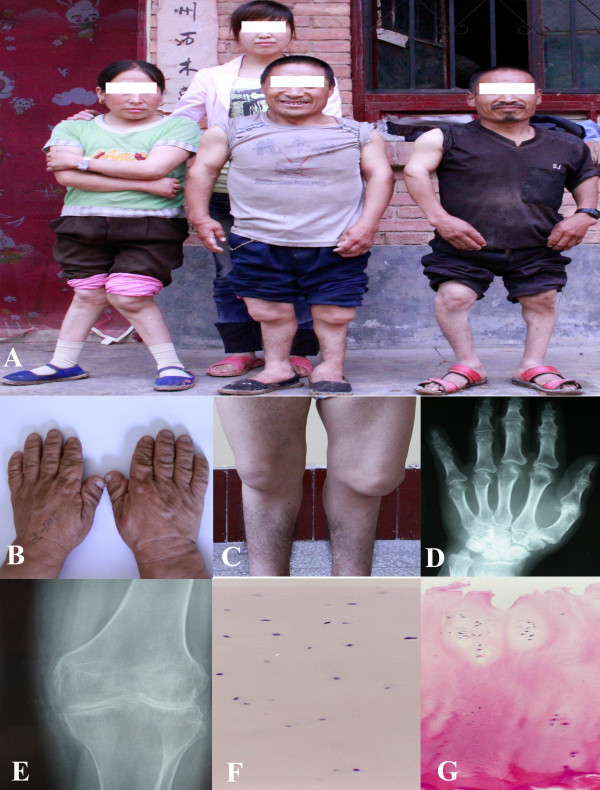
**Characteristics of Kashin-Beck disease (KBD)**. **A**, Representative KBD patients with grade III KBD(left female patient aged 43 years, middle male patient aged 49 years, right male patient aged 46 years); B&C, Images of hands(**B**) and knees (**C**) from representative patients with grade III KBD; D&E radiographic images of left hand(**D**) and right knee(**E**) of representative patient exhibiting shortened phalanges, enlarged bone ends of phalanges and narrowed joint space; F&G, hematoxylin and eosin staining of adult articular cartilage from a healthy subject (**F**) and KBD patients (original magnification × 100).

Articular cartilage specimens were collected from 9 adult KBD patients and 9 adult normal controls, respectively (Table 1). All study subjects were Chinese Han. There were 100 randomly selected patients with serious KBD undergoing free knee replacement surgery every year at Shaanxi province of China. Our 9 KBD patients were randomly selected from the KBD patients undergoing free knee replacement surgery, and came from the KBD prevalent areas-Linyou county and Yongshou county at Shaanxi province. According to the KBD clinical diagnosis criteria of China (Diagnostic code GB16395-1996), KBD patients were diagnosed with grade II or III KBD based on the radiography and cartilage sections after hematoxylin and eosin (H&E) staining(Figure 1) [15]. The healthy cartilage was collected from the knees of fresh cadaver within 8 hours of death caused by traffic accidents. All cadaver donors came from non-KBD prevalent areas, and excluded KBD, genetic bone and cartilage diseases, osteoarthritis and rheumatoid arthritis by cartilage section examination with H&E staining. The cartilage specimens were collected from the same anatomic area of femoral condyles of knee. The obtained cartilage specimens were rapidly dissected and frozen in liquid nitrogen, and stored at −80°C until RNA extraction.

**Table 1:** Characteristics of KBD and control sample pairs

	**KBD**		**Control**	
	**Age(years)**	**Sex**	**Age(years)**	**Sex**
Microarray sample set	55	Male	55	Male
	42	Male	37	Male
	52	Male	54	Male
	49	Female	48	Female
qRT-PCR sample set	69	Male	60	Male
	50	Male	56	Male
	51	Male	58	Male
	44	Female	34	Female
	57	Female	58	Female

### RNA preparation

Frozen cartilage specimens were rapidly ground in liquid nitrogen with freezer mill (SPEX CertiPrep, Metuchen, NJ, USA). Using Agilent Total RNA Isolation Mini kit (Agilent Technologies, Santa Clara, CA, USA), total RNA was isolated from cartilage following manufacturer recommended protocol. The quality and integrity of isolated total RNA were evaluated by 1% agarose gel electrophoresis.

### Microarray hybridization

The isolated total RNA was first transcribed into aRNA using Amino Allyl MessageAmp aRNA Kit (Applied Biosystems, Austin, TX, USA). The aRNA of KBD patients was labeled with Cy5 fluorescent dye, and the aRNA of healthy controls was labeled with Cy3 fluorescent dye. For each KBD-control pair, 0.5 μg of labeled aRNA was purified separately and mixed together with hybridization buffer before microarray hybridization. The hybridization solution was prepared using In Situ Hybridization Kit Plus (Agilent Technologies). Agilent Human 1A 22 k Whole Genome microarray (G4110B) that contains 22,575 oligonucleotides probes representing 21,073 human genes, was applied in this study. Microarray hybridization was performed in Gene-Machines hybridization chamber (Gene-Machines, San Carlos, CA, USA) following by washing according to Agilent oligonucleotide microarray hybridization protocol. Hybridization signals were recorded by Agilent scanner (G52565BA), and analyzed by Feature Extraction 9.3 (Agilent Technologies) and Spotfire 8.0 (Spotfire Inc., Cambridge, MA, USA) software. The quality of the fluorescent spots on the microarray was evaluated and recorded as present or absent. The fluorescent spots that failed to pass the quality control procedure were excluded for further analysis. Linear and LOWESS normalization were conducted to eliminate possible dye-related bias of the microarray data. The generated files were imported into spreadsheets (Excel, Microsoft Corp., Redmond, WA, USA) for following statistical analysis. Our microarray data are MIAME compliant and have been deposited in a MIAME compliant database ArrayExpress(Accession number:E-MEXP-3196).

### Data analysis

To evaluate the expression levels of MRG in KBD cartilage, 1,167 MRG were derived from environmentally related genomic database Toxicogenomics (http://ctd.mdibl.org/)[16,17]. The expression ratios of the 1,167 MRG were calculated from the microarray data. Significantly differently expressed MRG were defined by expression ratios <0.5 or >2.0.

To further investigate MRG expression patterns of KBD in the context of molecular functions and biological processes, gene set enrichment analysis software was used to identify differently expressed gene ontology(GO) between KBD cartilage and healthy cartilage [13,14]. GSEA is a computational method, which can be used to determine whether a set of functionally related genes present similar expression pattern between case and control groups. GSEA calculates a normalized enrichment score (NES) for each gene set, which reflects the over-represented degree of corresponding gene set in cases compared to controls. The obtained positive and negative NES values indicated the gene set up-regulation and down-regulation in KBD cartilage compared to healthy cartilage, respectively. The gene sets with extreme ES values were suggested to be significantly correlated with study phenotypes by GSEA[13,14]. Gene ontology database 3.0 containing 1,454 GO categories were downloaded from GSEA Website(http://www.broadinstitute.org/gsea/index.jsp), and applied in this study. Significantly differently expressed GO were defined as p values ≤ 0.05 calculated by GSEA.

### qRT-PCR validation

qRT-PCR was conducted to validate our microarray data using an independent sample set (Table 1). 4 up-regulated and 4 down-regulated genes in microarray experiment were randomly selected for qRT-PCR, including TMSL8, CASP8AP2, PAPSS2, VEGF, POSTN, TACC1, CBR3 and BMF. Total RNA was isolated and prepared in the same way as used by microarray experiment. Superscript II reverse transcriptase (Invitrogen, Carlsbad, CA, USA) was used to convert the isolated total RNA into cDNA. ABI 7500 Real-Time PCR Detection System (Applied Biosystems, Foster City, CA) was applied for amplification and detection of cDNA following manufacturer recommended protocol. Glyceraldehyde-3-phosphate dehydrogenase (GAPDH) was simultaneously assayed by qRT-PCR as an endogenous invariant control. All primer and probe sets were supplied by TaqMan Gene Expression Assays (Applied Biosystems). The expression levels of the 8 genes were normalized to the amount of GAPDH.

## Results

### Single gene expression analysis

Microarray experiment detected about 55%(mean ± SD 11,928 ± 1134) of all probe sets corresponding to the transcripts recognized as present in each cartilage sample. Small difference was observed between the percentage of transcripts expressed in KBD cartilage and that in healthy cartilage, KBD 53.9 ± 4.6% vs healthy controls 51.8 ± 8.1%.

As shown by Table 2, we identified 10 up-regulated MRG in KBD with an averaged expression ratio 5.23. The 10 up-regulated genes involve in various biological processes, including apoptosis, metabolism, extracellular matrix, growth factor and cytoskeleton & cell movement. Additionally, metabolism-related FABP4 and growth factor-related POSTN showed lower expression levels in KBD cartilage compared to healthy cartilage.

**Table 2:** Differently expressed MRG between KBD cartilage and healthy cartilage

**MRG**^**a**^	**ID**	**Mycotoxin**^**b**^	**Ratio**^**c**^
***Apoptosis***			
BAX	NM_138764	T-2 Toxin, Deoxynivalenol, Aflatoxin B1, Zearalenone	3.79 ± 1.53
BCL2	NM_000633	Deoxynivalenol	3.23 ± 0.97
***Extracellular matrix***			
COL5A2	NM_000393	Aflatoxin B1	8.85 ± 6.83
THBS1	NM_003246	Aflatoxin B1, Zearalenone	2.95 ± 0.76
***Metabolism***			
PDE8B	NM_003719	Aflatoxin B1	8.47 ± 4.08
GSTT2	NM_000854	Aflatoxin B1, Ochratoxin A	4.01 ± 1.51
FABP4	W60781	Aflatoxin B1	0.38 ± 0.09
***growth factor***			
IGFBP2	NM_000597	Aflatoxin B1	9.70 ± 3.73
IGFBP4	NM_001552	Aflatoxin B1	4.13 ± 1.53
POSTN	NM_006475	Aflatoxin B1	0.27 ± 0.12
***Cytoskeleton & cell movement***			
TMSL8	NM_021992	Aflatoxin B1	9.65 ± 8.50
TUBB2A	NM_001069	Fumonisin B1	3.27 ± 0.70
***Miscellaneous***			
VGLL3	NM_016206	Aflatoxin B1	4.10 ± 1.83
SOCS3	NM_003955	Deoxynivalenol	3.01 ± 0.71
FER1L3	NM_013451	Aflatoxin B1	2.82 ± 0.61

^a^ denotes mycotoxin-related genes.

^b^ defined by environmentally related genomic database Toxicogenomics.

^c^ expression ratio is presented as mean ± standard error of mean.

### Gene set expression analysis

GO expression analysis results are presented in Table 3. GSEA detected significant up-regulation of 4 apoptosis-related GO in KBD cartilage, including APOPTOSIS_GO (NES = 0.551), ANTI_APOPTOSIS(NES = 0.450), REGULATION_OF_PROGRAMMED_CELL_DEATH (NES = 0.470) and REGULATION_OF_APOPTOSIS (NES = 0.470) (Additional file 1: Figure S1). Additionally, 5 development-related GO presented higher expression levels in KBD cartilage than in healthy cartilage, including ORGAN_MORPHOGENESIS(NES = 0.663), ORGAN_DEVELOPMENT(NES = 0.511), SYSTEM_DEVELOPMEN(NES = 0.512), ANATOMICAL_STRUCTURE_DEVELOPMENT(NES = 0.506) and REGULATION_OF_DEVELOPMENTAL_PROCESS(NES = 0.473) (Additional file 2: Figure S2).

**Table 3:** Differently expressed gene ontology between KBD cartilage and healthy cartilage

**Gene Ontology Category**	**Function**	**NES**^**a**^	**P-value**
APOPTOSIS_GO	Apoptosis	0.450	0.032
REGULATION_OF_APOPTOSIS	Apoptosis	0.470	0.032
REGULATION_OF_PROGRAMMED_CELL_DEATH	Apoptosis	0.470	0.032
ANTI_APOPTOSIS	Apoptosis	0.551	<0.001
ORGAN_DEVELOPMENT	Development	0.511	<0.001
SYSTEM_DEVELOPMENT	Development	0.512	<0.001
REGULATION_OF_DEVELOPMENTAL_PROCESS	Development	0.473	0.050
ORGAN_MORPHOGENESIS	Development	0.663	<0.001
ANATOMICAL_STRUCTURE_DEVELOPMENT	Development	0.506	<0.001

^**a**^ denotes normalized enrichment score (NES) calculated by GSEA. Positive NES indicate gene ontology up-regulated in KBD cartilage compared to healthy cartilage.

### qRT-PCR validation

**Figure 2:**
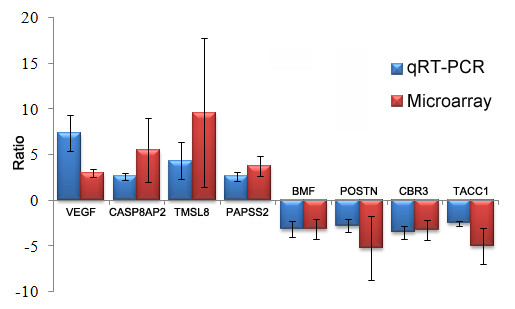
Histogram showing the expression values of the selected 8 genes measured by microarray and qRT-PCR.

As shown by Figure 2, we observed increased expression levels of TMSL8, CASP8AP2, PAPSS2 and VEGF in KBD cartilage compared to healthy cartilage. The expression levels of POSTN, TACC1, CBR3 and BMF appeared to decrease in KBD cartilage. The expression patterns of the 8 genes were consistent between microarray and qRT-PCR, confirming the validity of our microarray data.

## Discussion

In this study, we compared MRG expression profiles between KBD cartilage and healthy cartilage, and identified a set of differently expressed MRG and GO. Based on the results of previous and our studies, we suggested that mycotoxins might contribute to the cartilage lesions of KBD through affecting the expression and biological function of MRG involved in apoptosis, collagen synthesis and growth & development in cartilage.

### Apoptosis

Excess chondrocyte apoptosis is one of the primary pathological changes of KBD [1,18]. The molecular mechanism underlying abnormal chondrocyte apoptosis in KBD remains unclear now. In this study, we found that apoptosis-related BAX and BCL2 genes were significantly up-regulated in KBD. This result is consistent with that of previous study, which observed excess chondrocyte apoptosis and increased expression of BAX and BCL2 in KBD cartilage compared to healthy cartilage. GO expression analysis further found that 4 apoptosis-related GO were significantly up-regulated in KBD cartilage. BAX and BCL2 are important apoptosis regulatory factors in human body [19]. According to the environmentally related genomic database Toxicogenomics (http://ctd.mdibl.org/), the expression and biological function of BAX and BCL2 suffer from the impact of multiple mycotoxins. For instance, it was reported that T-2 toxin was able to induce apoptosis via BAX and BCL-2 mediated apoptosis process [8,20]. Because T-2 toxin was an important environmental risk factors of KBD [1,5], we may infer that T-2 toxin contributed to the cartilage lesions through dysfunction of BAX/BCL2 mediated apoptosis in KBD. Further studies may be necessarily to investigate the role of BAX/BCL2 mediated apoptosis in KBD cartilage damages caused by mycotoxins.

### Extracellular matrix

Previous study found that mycotoxins could damage collagen in KBD cartilage [21], but the molecular mechanism was not clear. In this study, we found that collagen gene COL5A2 was significantly up-regulated in KBD cartilage. COL5A2 encodes an alpha chain of type V collagen, which is a minor component of extracellular matrix. It was reported that type V collagen could regulate the initiation of collagen fibril assembly [22]. The lack of type V collagen results in reduction of collagen production and abnormality of collagen structure [22], which were observed in KBD cartilage [23]. Another study found that mycotoxins could alter the expression of COL5A2 in human body [24], which might lead to abnormal collagen production [23]. Therefore, we may infer that the dysfunction of type V collagen regulated collagen production contributed to mycotoxins-induced cartilage lesions in KBD.

We observed that THBS1 gene was significantly down-regulated in OA cartilage compared to KBD cartilage. Another study also found that THBS1 was significantly down-regulated in OA compared to rheumatoid arthritis [25]. It seems that THBS1 might be a biomarker for distinguishing OA from KBD and rheumatoid arthritis. THBS1 encodes thrombospondin 1, which is an adhesive glycoprotein and can mediate cell-to-cell and cell-to-matrix interactions. Previous study found that THBS1 protein could bind to type V collagen, and played an important role in angiogenesis [26]. Furthermore, Pasteels JL et.al suggested that abnormal angiogenesis contributed greatly to the development of KBD [27]. Hinsenkamp M et.al suggested that mycotoxins might contribute to the occurrence of KBD through inhibition of angiogenesis [28]. Given our study results, the role of THBS1 in the development of KBD may be worthwhile for further studies.

### Growth and development

Serious KBD patients usually have skeletal developmental disorders, including short fingers, short limb and short stature. In this study, development-related IGFBP2 and IGFBP4 genes presented higher expression levels in KBD cartilage than in healthy cartilage. The proteins encoded by IGFBP 2 and IGFBP4 belong to insulin-like growth factor binding protein family, which can inhibit growth and development through binding to insulin-like growth factor [29,30]. Previous microarray study found that mycotoxins were able to alter the expression of IGFBP2 and IGFBP4 [24]. Additionally, GO expression analysis observed that 5 development-related GO were significantly up-regulated in KBD cartilage. Mycotoxins may contribute to the skeletal developmental disorders of KBD through affecting the expression and biological function of growth and development-related genes, such as IGFBP2 and IGFBP4.

## Conclusions

We investigated the MRG expression profiles of KBD, and identified a set of differently expressed MRG and GO between KBD cartilage and healthy cartilage. Based on the results of previous and our studies, we suggest that mycotoxins might contribute to the development of KBD through dysfunction of MRG involved in apoptosis, collagen synthesis and growth & development in cartilage. Our efforts may help to understand the molecular mechanism underlying KBD.

## Competing interests

The authors declare that they have no competing interests.

## Authors’ contributions

FZ and XG designed the study. WZW and XG prepared all test samples and conducted the experiments. FZ and SXW performed statistical analysis and prepared the manuscript. All authors have read and approved the final manuscript.

## Pre-publication history

The pre-publication history for this paper can be accessed here:

http://www.biomedcentral.com/1471-2474/13/130/prepub

## Supplementary Material

Additional file 1 **Figure S1. GSEA gene ontology expression analysis results of APOPTOSIS_GO (A), REGULATION_OF_APOPTOSIS(B), REGULATION_OF_PROGRAMMED_CELL_DEATH (C) and ANTI_APOPTOSIS (D).** The top portion of each plot denotes the running enrichment score(ES), which reflects the overrepresented degree of corresponding gene set. The positive and negative ES values indicate gene set up-regulation and down-regulation in KBD compared to healthy controls, respectively. The middle portion of each plot shows the place of members of the gene set in the ranked list of genes. The bottom portion of each plot shows the values of ranking metric as it moving down the ranked list of genes. Click here for file

Additional file 2 Figure S2. GSEA gene ontology expression analysis results of ORGAN_DEVELOPMENT (A), SYSTEM_DEVELOPMENT (B), REGULATION_OF_DEVELOPMENTAL_PROCESS (C), ORGAN_MORPHOGENESIS (D) and ANATOMICAL_STRUCTURE_DEVELOPMENT (E). Click here for file
