# Supplementary figures and images for: Expression profile analysis of mycotoxin-related genes in cartilage with endemic osteochondropathy kashin-beck disease
Source: BMC Musculoskelet Disord. 2012 Jul 24;13:130. doi: 10.1186/1471-2474-13-130 (PMC3416648; doi:10.1186/1471-2474-13-130)

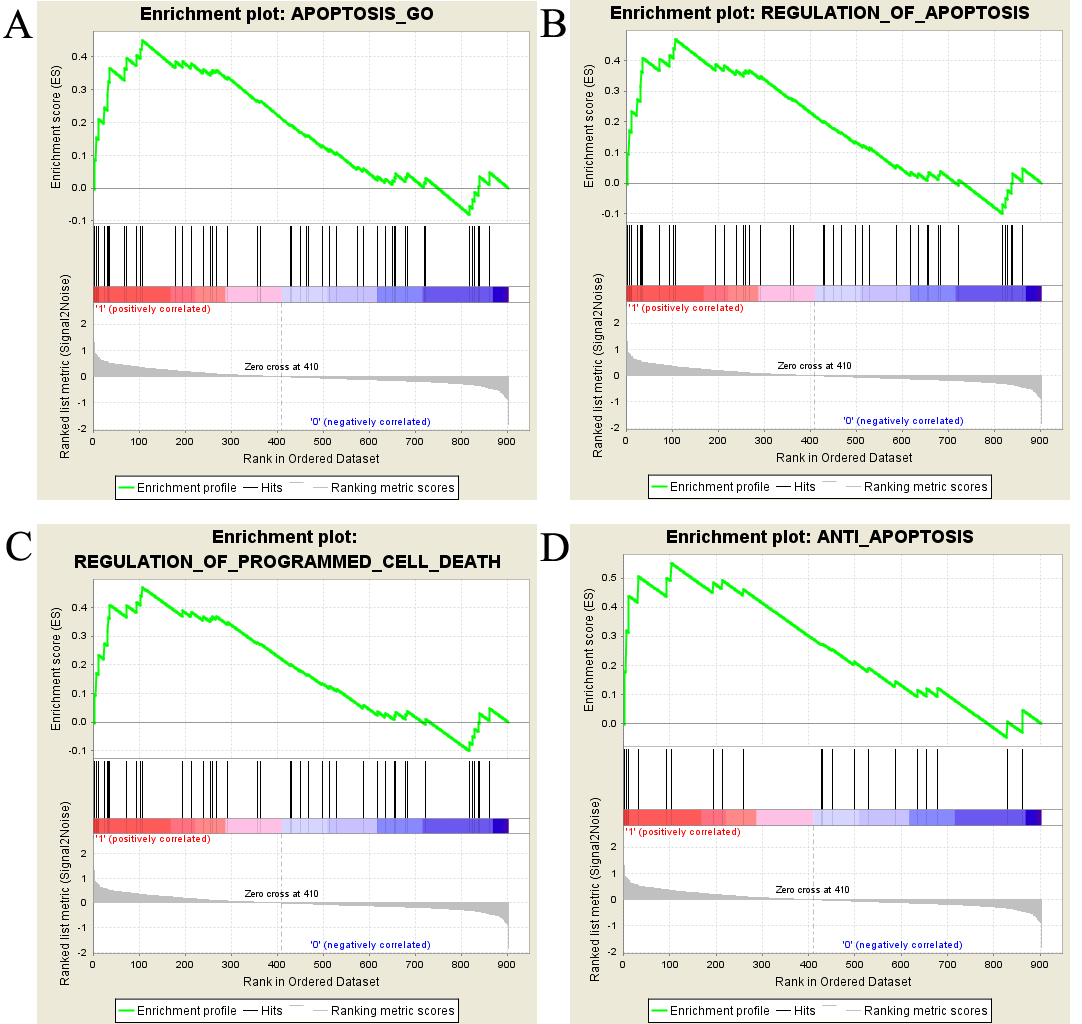

Supplement: Additional file 1 — Figure S1. GSEA gene ontology expression analysis results of APOPTOSIS_GO (A), REGULATION_OF_APOPTOSIS(B), REGULATION_OF_PROGRAMMED_CELL_DEATH (C) and ANTI_APOPTOSIS (D). The top portion of each plot denotes the running enrichment score(ES), which reflects the overrepresented degree of corresponding gene set. The positive and negative ES values indicate gene set up-regulation and down-regulation in KBD compared to healthy controls, respectively. The middle portion of each plot shows the place of members of the gene set in the ranked list of genes. The bottom portion of each plot shows the values of ranking metric as it moving down the ranked list of genes. [file 1471-2474-13-130-S1.tiff]

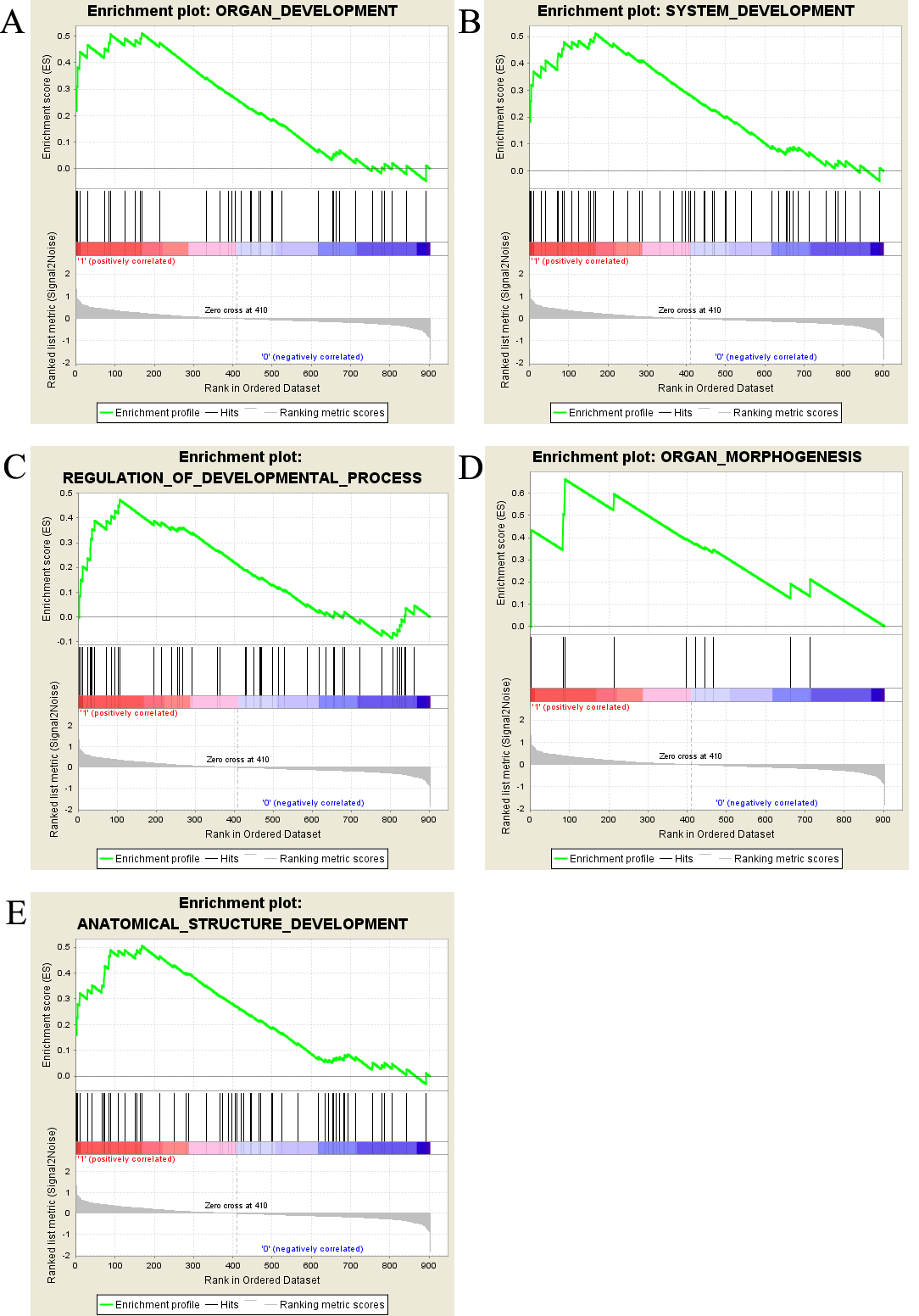

Supplement: Additional file 2 — Figure S2. GSEA gene ontology expression analysis results of ORGAN_DEVELOPMENT (A), SYSTEM_DEVELOPMENT (B), REGULATION_OF_DEVELOPMENTAL_PROCESS (C), ORGAN_MORPHOGENESIS (D) and ANATOMICAL_STRUCTURE_DEVELOPMENT (E). [file 1471-2474-13-130-S2.tiff]
